# Efficient removal of humic acid in water using a novel TiO_2_ composite with biochar doping

**DOI:** 10.1039/d2ra05358f

**Published:** 2022-11-08

**Authors:** Guoqiao Wang, Jiawei Wang, Tian Yu, Xin Guo, Yao Chen

**Affiliations:** College of Architecture and Environment, Sichuan University Chengdu 610065 China chenyao@scu.edu.cn +86-28-8540-5613 +86-28-8540-3016; Shenyang Municipal Bureau of Public Utilities Shengyang 110011 China; Chengdu Academy of Environmental Protection Sciences Chengdu 610072 China

## Abstract

Titanium dioxide modified with biochar (Ti–C) was prepared by a sol–gel method for the degradation of humic acid (HA) in aqueous solutions. Under identical conditions, Ti–C contained less TiO_2_ and showed better HA degradation capacity than that of pure TiO_2_, and had *ca.* 20% higher HA removal rate than that of simple Ti–C adsorption. Photocatalytic degradation of HA with Ti–C had an efficient removal rate of 50% at pH = 3, which was *ca.* 28% higher than that at pH = 7 (HA = 10 mg L^−1^), while a higher reaction temperature, longer lighting time and larger Ti–C dosage were conducive to HA photocatalytic degradation. SEM micrographs showed that Ti–C had a much rougher surface than the original biochar, and EDS results of Ti–C indicated that its carbon content increased up to 26.2% after biochar doping. Ti–C had an evident anatase structure and a typical SiO_2_ structure, as revealed by XRD analysis. TOC and GC-MS analysis indicated that HA was effectively degraded and transformed into harmless carbon dioxide. Superoxide radicals were the main active radicals produced for the efficient degradation of humic acid, while hydroxyl radicals and electron–holes also contributed to HA decomposition in Ti–C systems. This work is expected to be helpful for the innovative preparation of titanium dioxide as a low-cost photocatalyst for the degradation of humic acid in water.

## Abstract 2

Titanium dioxide modified with biochar (Ti–C) was prepared by a sol–gel method for the degradation of humic acid (HA) in aqueous solutions.

## Graphical abstract

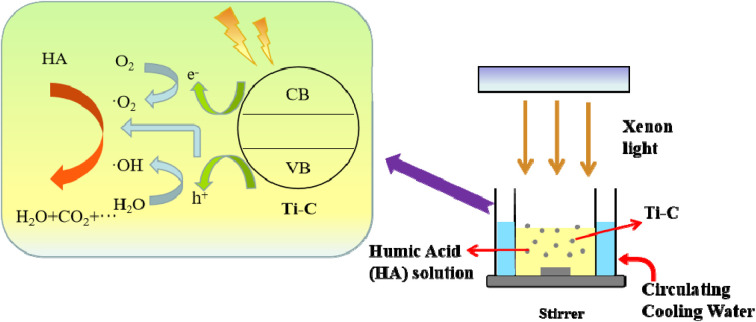

## 1 Introduction

With the rapid development of industries, agriculture, forestry, animal husbandry and aquaculture, as well as the continuous growth of population and improvement of people's living quality, environmental pollution by wastewater discharge containing various organic compounds has become increasingly serious.^1^ Organic compounds are widely distributed in nature, which is the key factor causing a series of problems such as odor and water quality damage. Carboxyl and hydroxyl groups are typical organic material's molecules, which can react with most pollutants. They could highly affect the morphological transformation, biological toxicity and effective degradation of those pollutants, and will be a serious threat to the ecological environment and biological health.^2–4^

Humic acid (HA) is one of the most common organic compounds, which exists widely in nature.^5^ Excessive HA is proved to cause severe water pollution and could form certain carcinogens such as disinfection by-products (DBPs) and trihalomethanes (THMs) during chlorination and disinfection in water treatment plants (WTPs).^6^ Extensive research has demonstrated that HA will chelate with metal ions, thus making it unfavorable to treat heavy metal ions.^7^ HA can further enhance the stability of colloidal particles and reduce the coagulation effect. It will also interfere in absorption processes to produce toxic substances.^8^ Studies showed that humic acid was likely to cause Kashin–Beck disease (KBD). The content of humic acid in Kashin–Beck disease-affected areas was significantly higher than that in unaffected areas.^9^

Currently, methods focused on humic acid removal mainly include physical (adsorption, membrane separation, and enhanced coagulation),^10^ chemical (ozone oxidation, photoelectric oxidation, chemical oxidation, and photocatalytic oxidation),^11^ and biological methods (anaerobic or aerobic biological treatment). Photocatalytic oxidation is one of the most efficient and simple ways to remove pollutants in aqueous solutions.^12,13^ This is superior to organic matter degradation due to its efficiency and convenience.^14,15^ Titanium dioxide (TiO_2_) with great photocatalytic activity and good practicality is one kind of representative photocatalysts.^16^ Furthermore, TiO_2_ specifically doped with certain elements is much more conducive to HA degradation.^17^ However, high cost of preparation and hard separation from water are two drawbacks limiting the development and utilization of titanium dioxide.

To enhance titanium dioxide's optical response and reduce its recombination probability of photogenerated electrons and holes, modification is suggested to improve TiO_2_ performance and thus to effectively degrade organic compounds from the solution. With special modifiers, titanium dioxide could decompose organic compounds from wastewaters rapidly and thoroughly.^18^ TiO_2_ composed of adsorbents could effectively accumulate pollutants nearby, and thus, a high concentration of pollutant environment can be formed around the photocatalyst, accelerating the reaction process. Extensive research has been carried out in this field.^19,20^

Biochar is a carbon-rich solid formed by the pyrolysis of biomass at a high temperature under anaerobic or anoxic conditions. Biochar has a high specific surface, abundant pore structures and a large number of functional groups, which are favorable for the adsorption of heavy metals, organic pollutants and other contaminants.^21^ There are a wide range of raw materials such as plant roots, sawdust, wheat straw, and animal manure to prepare biochar.^22,23^ As an economic natural substance and a good additive agent, biochar is used to improve the degradation capacity of titanium dioxide. Environmental friendliness, rich resources and easy regulation of structural properties are great strengths for biochar prepared from waste-biomass pyrolysis. Sewage sludge produced in the urban wastewater treatment process is rich in carbonaceous materials, which has been successfully used to prepare economic and applicable biochar.

TiO_2_ loaded with sludge-based biochar has the advantage of synthetic actions both from adsorptive carbon-based materials and photocatalytic properties of TiO_2_. Sludge-based biochar provides more reaction sites for TiO_2_, which are helpful for enhancing the surface activity of TiO_2_.^24^ Biochar doping also improves the electron transfer rate and broadens the optical response on TiO_2_, and strengthens the synergistic effect between carbon and titanium elements. Actually, there is limited information on humic acid degradation using biochar-doped titanium dioxide.

In this study, sludge-based biochar was prepared and doped to titanium dioxide by a sol–gel method to enhance HA uptake in solutions. The effectiveness of this modified titanium dioxide was verified by adsorption and photocatalytic experiments. The impact of the reaction time, initial pH, reaction temperature and dosage on the Ti–C photocatalytic removal of HA was discussed and analyzed. Physicochemical characteristics including surface morphology, elemental composition, and crystal structure of the composed product were investigated *via* SEM-EDS and XRD analyses. The degradation and transformation of humic acid molecules during the reaction were investigated using a TOC and GC-MS monitor, and the performance of various active free radicals during photocatalysis was investigated *via* quenching experiments by adding different radical scavengers. It would be helpful for humic acid removal from solutions and better understanding the photocatalytic reaction of this composed photocatalyst in wastewater treatment.

## 2 Materials and methods

### 2.1 Ti–C photocatalyst preparation

Sewage sludge used as the raw material to prepare the biochar was collected from a local wastewater treatment plant in Chengdu. Sulfuric acid (H_2_SO_4_) and zinc chloride (ZnCl_2_) were used as activating agents to handle sludge. The activated sludge was carbonized to form biochar at a high temperature (550 °C) in a N_2_ atmosphere (200 mL min^−1^). After cooling down and washing with deionized water to get the final elution at pH = 7.0, the produced biochar was dried for further modification. The details of biochar production were in accordance with our previous research.^25^

First, 7.2 mL butyl titanate mixed with 20 mL ethanol was shaken at 450 rpm for 0.5 h. Then, hydrochloric acid was carefully added to adjust the solution pH to about 3.0 during the whole operation and oscillated for 0.5 h. After that, 0.2 g biochar powder was added into the solution and stirred for another 0.5 h (450 rpm) to get thorough mixing. Subsequently, 10 mL of 44% ethanol was added to this solution and stirred continuously until gel formation occurs.

Then, the wet gel was kept airtight, aged for 24 h and dried immediately in an oven at 100 °C for 12 h to obtain xerogel. The xerogel was fully ground into a powder and heated in the tubular furnace at a constant heating rate of 2 °C min^−1^ to a temperature of 500 °C in a nitrogen atmosphere. After 4 h of reaction at 500 °C, the finished product was cooled down to room temperature, washed with deionized water and then dried to obtain final composed TiO_2_ with biochar modification, which was designated Ti–C. Pure titanium dioxide (TiO_2_) was obtained without the addition of biochar during the same preparation processes.

Sulfuric acid, zinc chloride, commercial titanium dioxide (anatase-TiO_2_), butyl titanate, ethanol, hydrochloric acid, isopropanol, ammonium oxalate and formic acid used in the experiments are of analytical purity (AR). Humic acid is a refined natural mixture purchased from RHAWN (CAS: 1415-93-6).

### 2.2 Photocatalytic experiment

Photocatalytic experiments were carried out to better understand the performance of Ti–C on humic acid (HA) degradation under xenon light irradiation. A high-pressure xenon lamp (PL-XQ 500 W) was used as the light source (light tube powder: 25 A; irradiation: AM 1.5 simulated sunlight 0–2500 nm wavelength). A HA solution (10 mg L^−1^) was prepared by dissolving humic acid in a certain amount of deionized water. The experiments were conducted in a series of 200 mL beakers containing 100 mL humic acid solution with a magnetic stirrer (450 rpm). The temperature of the reaction system was kept constant (15 °C) by an external recirculating cooling water system. The distance between the lamp and the surface of the solution in the photocatalytic system was 8 cm. Water samples were collected at desired time intervals and filtered through a 0.22 μm micron water filtration membrane. The supernatant was analyzed to determine the HA concentration. All experiments were carried out in duplicate, and the average values were reported.

The removal rate of humic acid in the solution was calculated using eqn (1):1
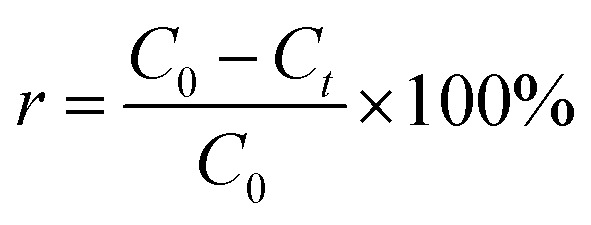
where *r* is the humic acid removal rate, *C*_0_ is the humic acid initial concentration, and *C*_*t*_ is the humic acid concentration at time *t* (mg L^−1^).

**Fig. 1:**
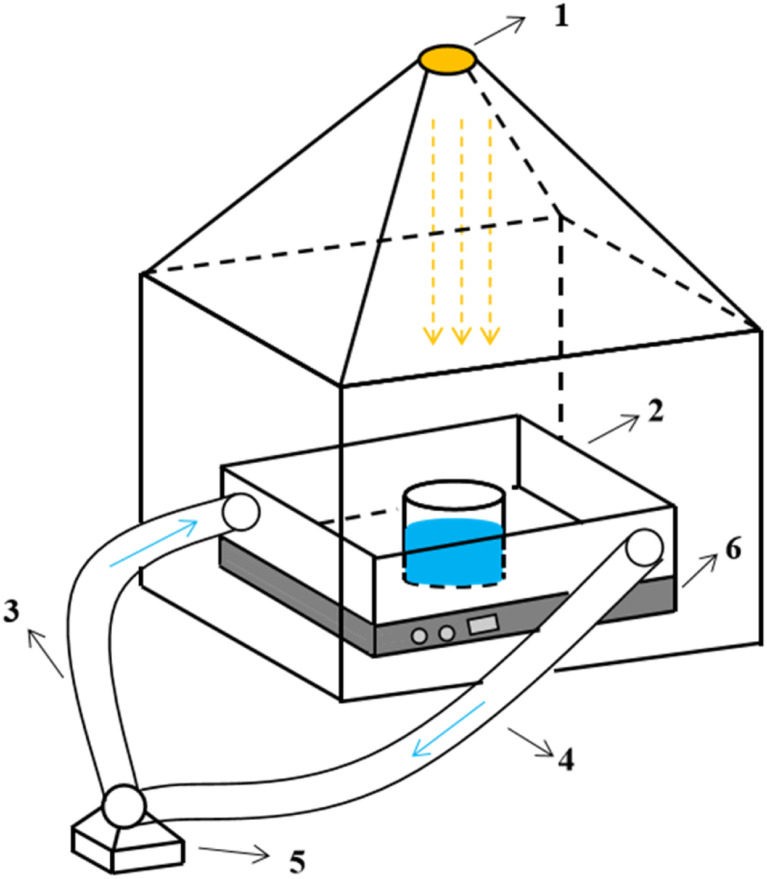
Schematic of the experimental set-up ((1) light source; (2) reaction system; (3) water inlet system; (4) effluent system; (5) circulating pump; (6) stirrer).

The schematic of the experimental set-up is shown in Fig. 1.

### 2.3 Characterization

Ultimate analysis and surface morphology of the produced biochar and Ti–C (Scanning Electron Microscope-Energy Dispersive Spectrometer, SEM-EDS) were completed at the Analytical and Testing Center of Sichuan University using a JSM-7500F field emission scanning electron microscope (JEOL). The main operational parameters of the instrument are as follows: accelerating voltage of 0.1–30 kV; resolution of 1.0 nm (15 kV) and 1.4 nm (1 kV); and the magnification among ×25–×800000. X-ray diffraction, XRD (Empyrean, PANalytical B.V.) was used for phase identification with a Cu Kα radiation from 10° to 70°.

HA concentration of the supernatant was analyzed by a UV visible spectrophotometer (UV-1100, MAPADA), and the wavelength range was from 200 to 2000 nm. pH values were tested by a pH meter (PHB-4, Rex).

A total organic carbon analyzer (TOC, Analytik jena) was used to determine the content of organic matter in solution, so as to analyze the mineralization degree of organic matter.

GCMS-QP2010 Plus gas chromatography-mass spectrometry (GC-MS, Shimadzu Co. Ltd., Japan) was performed to determine the relative content of organic components in the solution, so as to analyze the degradation of organic substances.

### 2.4 Quenching experiment

Electron–holes, hydroxyl radicals and superoxide radicals with a strong redox ability would be produced during the photocatalytic process, and thus be utilized to degrade pollutants.^26^ Isopropanol (IPA),^27^ ammonium oxalate (AO),^14^ and formic acid (FA)^28^ were used to verify hydroxyl radicals, electron–holes and superoxide radicals, respectively. After equilibrium adsorption of humic acid on Ti–C, 10 mM IPA, 10 mM AO and 2 mM FA were added into a series of testing solutions (HA concentration: 10 mg L^−1^; Ti–C dosage: 1.0 g L^−1^; stirring rate: 450 rpm; initial pH: 7.2; temperature: 15 °C), respectively, to capture various active radicals. The HA concentration was monitored to learn the degrading inhibition of hydroxyl radicals, electron–holes and superoxide radicals at certain intervals.

## 3 Results and discussion

### 3.1 Adsorption and photocatalysis

The adsorption and photocatalysis performance of Ti–C on HA removal was carried out by adding 1 g L^−1^ of TiO_2_ or Ti–C into a series of 10 mg per L HA solutions put under dark and photocatalytic conditions, respectively. After the desired reaction time, solutions were filtered and sampled. The HA concentrations of the supernatant were analyzed using a UV visible spectrophotometer.

**Fig. 2:**
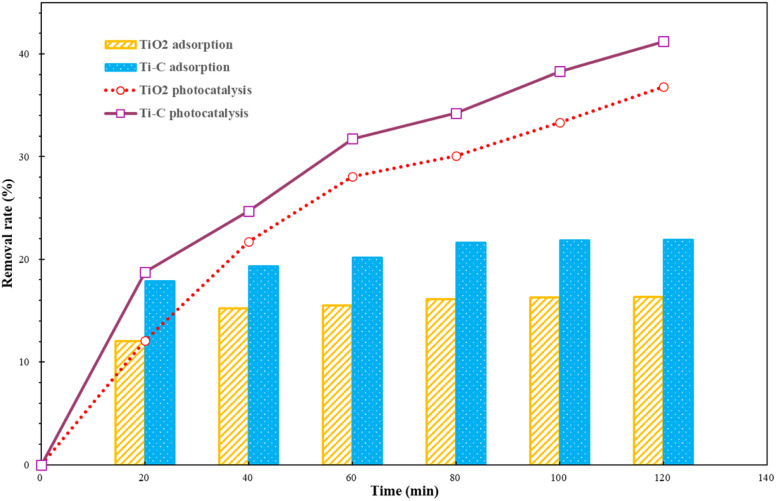
Humic acid adsorption and photocatalytic removal by TiO_2_ and Ti–C (HA: 10 mg L^−1^; TiO_2_: 1 g L^−1^; temperature: 15 °C; pH: 7.2).

Fig. 2 shows the adsorptive and photocatalytic performance of TiO_2_ and Ti–C in HA degradation. As shown in Fig. 2, Ti–C had better adsorption capacity than that of pure TiO_2_, which indicated that addition of biochar effectively improved the adsorption capacity of HA on the product. The HA removal rates of both materials in the first 20 minutes under lighting were similar to those in the dark reactions. This proved that HA elimination from the solution was dominated by adsorption at the initial stage for both materials. The HA removal rate on both materials without lighting changed slightly after 40 minutes, which showed the achievement of adsorption equilibrium. In the following experiments, photocatalysis experiments were all discussed based on adsorption equilibrium.

Both materials showed a higher HA removal rate with light irradiation compared to dark reactions. This displayed good photocatalytic ability on both materials, and much better HA degradation than simple adsorption. With the progress of the reaction, the photocatalytic removal rate of HA for both materials was increasing, which indicated that HA photocatalytic degradation was stable and persistent. After 2 h, the HA photocatalytic removal rate of both photocatalysts was almost twice higher than that under simple adsorption. Under identical conditions, Ti–C contained less TiO_2_ and presented better HA degradation capacity than that of pure TiO_2_. This proved that the addition of biochar not only improved Ti–C's adsorption capacity on HA, but also reduced the agglomeration of the photocatalyst. Doping biochar on titanium dioxide made the composite more available and economic for water pollutant degradation.^29^

Biochar had a high adsorptive ability on HA and the removal rate was 88.4% after 40 min to attain equilibrium, while this adsorbent was one-off and need regeneration after exhaustion. Though the adsorption capacity of Ti–C was much lower than that of biochar, the photocatalytic effect lasted during degradation for HA removal. The removal of humic acid by light degradation could be continued with the increase in reaction time and Ti–C would not be exhausted. It is much potentially and economically feasible to decompose HA simultaneously *via* adsorption and photocatalysis by Ti–C than *via* only adsorption by biochar.

### 3.2 Photocatalytic operation

First, 1 g per L Ti–C was added into 100 mL 10 mg per L HA solution, and the photocatalytic reaction was carried out after adsorption equilibrium (dark operation) to fully understand the change in HA degradation in the solution by the composite. After the desired lighting time, the samples were filtered out and HA concentrations of the supernatant were determined using a UV-vis spectrophotometer.

**Fig. 3:**
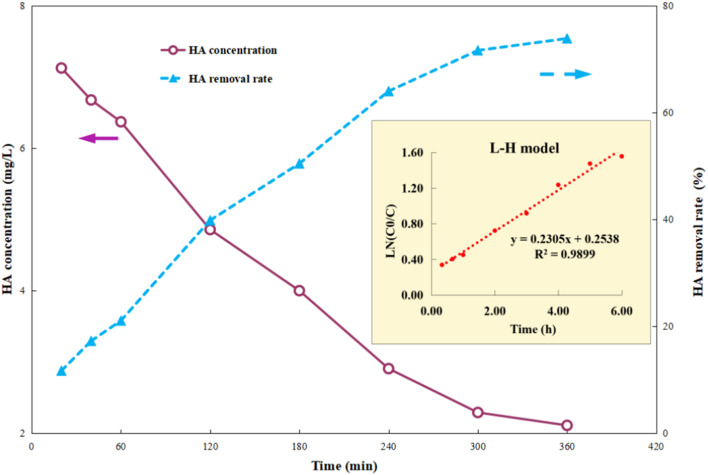
Humic acid photocatalytic removal by Ti–C at different reaction time points (HA: 10 mg L^−1^; TiO_2_: 1 g L^−1^; temperature: 15 °C; pH: 7.2).

Fig. 3 shows that HA removal by Ti–C continuously increases with the increment in lighting time. It was *ca.* 40% after 2 h photocatalytic reaction, indicating that the constituent and structure of HA in the solution changed rapidly in the photocatalytic system. The HA removal rate kept constantly increasing to 64% in the following 2 h, and there was insignificant change between 72 and 74% after 5–6 h lighting. Due to accumulation of intermediates and consumption of active free radicals, this system was stable with less than 2% change of HA degradation after a long-time operation. Those degradation intermediates were hard to decompose further, thus consequently affected the HA removal.

Photocatalysis proves to be a heterogeneous reaction. According to Langmuir's adsorption theory and mass action law, if molecules occupied a single position on the catalyst surface and were not separated, the photocatalytic reaction kinetics could be described by the Langmuir–Hinshelwood (L–H) equation after adsorbed molecules reached equilibrium,^30^ and the expression is described as eqn (2):2
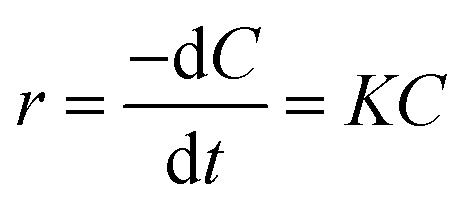
where *r* is the reaction rate, *C* is the HA concentration at time *t*, and *K* is the apparent first-order degradation constant of photocatalytic reaction. After numerical integration, it is changed to eqn (3):3
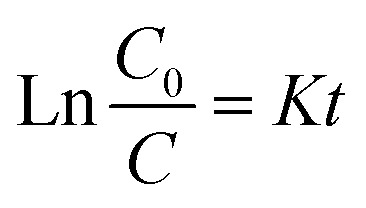
where *C*_0_ is the initial concentration of HA. As shown in Fig. 3, the correlation coefficient *R*^2^ was 0.9899 for the L–H model, indicating that photocatalytic degradation kinetics of Ti–C fitted well to the L–H model.

Table 1 shows the removal rate of Ti–C and other composite materials for photocatalytic degradation of humic acid. As listed in Table 1, Ti–C has much favorable photocatalytic degradation performance towards humic acid compared to others.

**Table 1:** Comparison of photocatalytic degradation of HA among Ti–C and other materials

Material	Removal rate (%)	Reference
TiO_2_-carbonaceous hyper-cross-linked polystyrene polymer	*ca.* 30%	11
Carbon nanotubes–TiO_2_-epoxy	43.8	24
0.6 wt% Fe–TiO_2_ supported on spherical activated carbon	*ca.* 35%	31
Ti–C	*ca.* 40%	This paper

The solution pH plays an important role during the process of photocatalytic oxidation, which has specific impacts on various pollutants. First, 1 g per L Ti–C was added into 100 mL of 10 mg per L HA solution, and its initial pH was around 7.2. The pH of the solution was adjusted between 3 and 11 by adding 1 M HCl or NaOH.

**Fig. 4:**
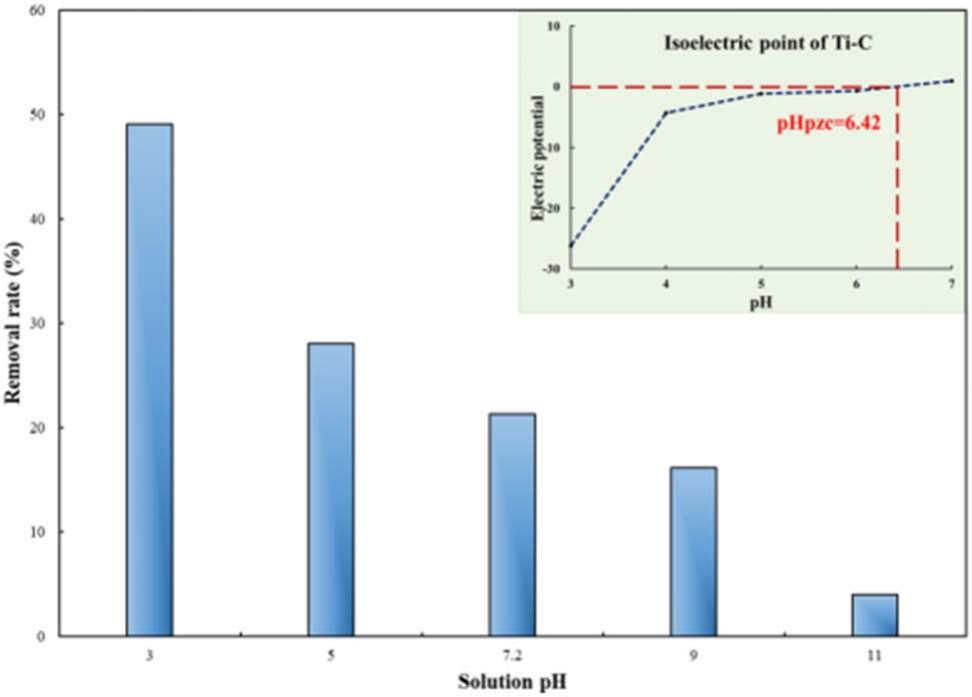
Humic acid photocatalytic removal by Ti–C at different solution pH values (HA: 10 mg L^−1^; TiO_2_: 1 g L^−1^; reaction time: 60 min; temperature: 15 °C).

As illuminated in Fig. 4, within the 3–11 pH range, the higher the pH value of the solution, the lower the HA removal on Ti–C due to the weak contact ability of humic acid molecules and Ti–C under alkaline conditions.^6^ The HA removal rate was around 50% at pH 3.0, 21.3% at initial pH (7.2) and only 4.0% at pH 11.0 in the solution. The removal rate obviously increased *ca.* 28% when the pH adjusted from 7.2 to 3.0, while it decreased 17.3% when the pH was adjusted from 7.2 to 11.0. HA molecule solubility in water was apparently affected by the solution pH. As the solution pH decreased, HA molecules would precipitate from the solution and the contact between HA and Ti–C was consequentially more sufficient and efficient. As shown in Fig. 4, the isoelectric point (pHpzc) of produced Ti–C was 6.42. The surface of the Ti–C composite was negatively charged when the solution pH was higher than its pHpzc, while the surface of humic acid was also negatively charged under alkaline conditions due to the existence of hydroxyl and carboxyl groups, which was unfavorable for its deposition on Ti–C, and then reducing HA removal rate. The surface of Ti–C was positively charged when the solution pH was lower than its pHpzc, and the surface of humic acid was neutral and helpful for adsorption, thus resulting in an increment in the HA removal rate. Furthermore, particulates were easy to agglomerate and form large particles in the solution when the pH was around their pHpzc. The dispersion of particles was highly improved when the pH was far away from their pHpzc, which were conducive to increasing the available area of illumination and light transmission, thus increasing the photocatalytic activity and HA degradation efficiency.^14,20^

First, 1 g per L Ti–C was added into 100 mL 10 mg per L HA solution at different temperatures to study the effect of temperature on this photocatalytic system.

**Fig. 5:**
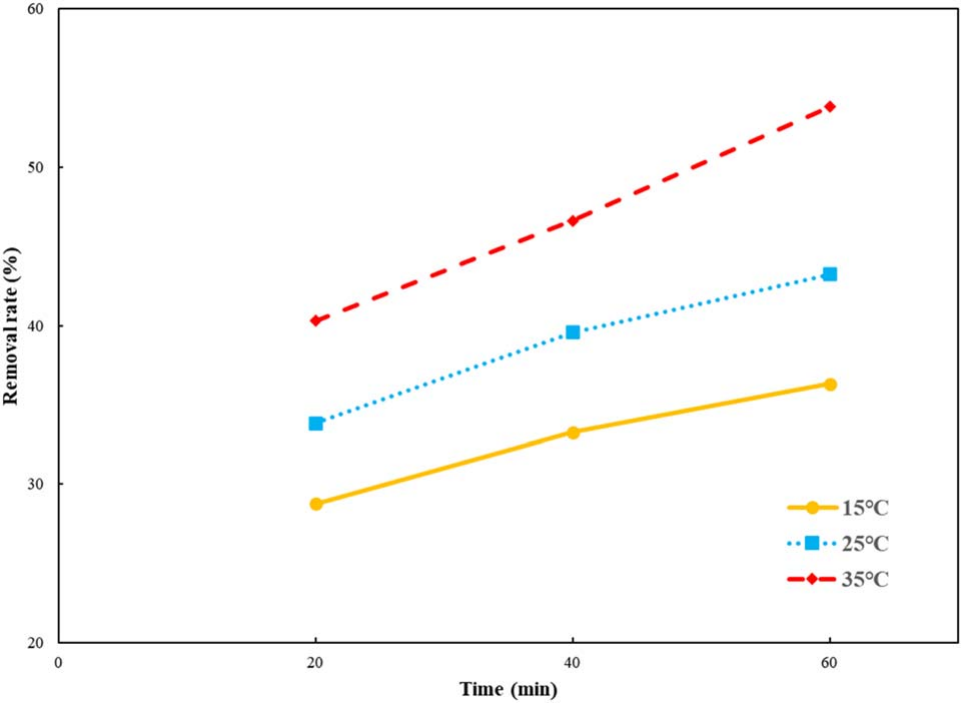
Humic acid photocatalytic removal by Ti–C at different reaction temperatures (HA: 10 mg L^−1^; TiO_2_: 1 g L^−1^; pH: 7.2).

As shown in Fig. 5, the HA removal rate increased with the increase in temperature. The light radiation was enhanced at a high temperature, which made Ti–C absorb more photon energy and produce more electrons and holes, as well as more active substances, thus allowed better HA degradation in the system. Temperature rise could also accelerate the mass transfer rate, including HA adsorption on the Ti–C surface and active substance diffusion into water, thereby promoting the removal rate.^32^

Ti–C (0.5–2 g L^−1^) was put into a series of 100 mL HA solutions (10 mg L^−1^) and mixed thoroughly (450 rpm) for 60 min after adsorption equilibrium (40 min dark reaction).

**Fig. 6:**
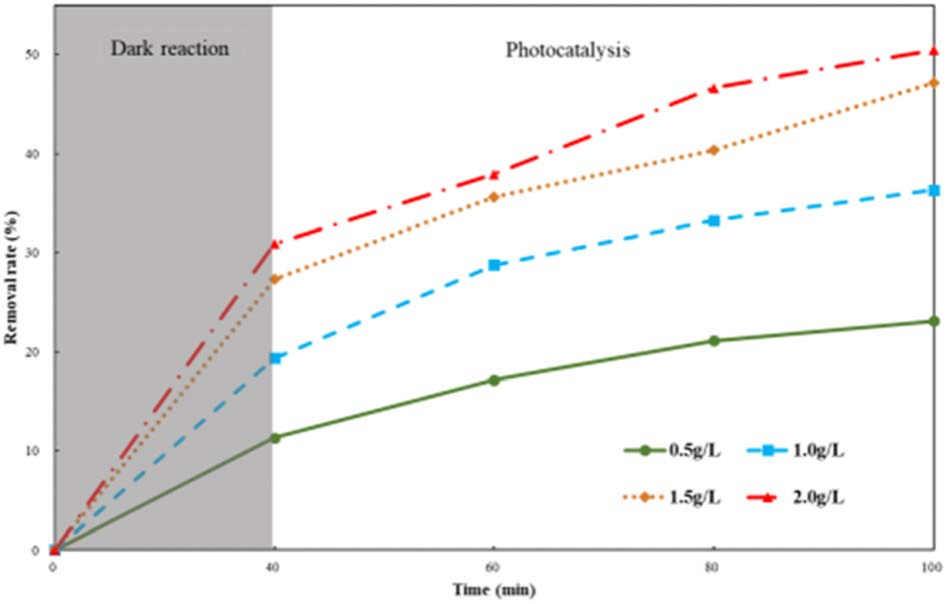
Humic acid photocatalytic removal with different Ti–C dosages (HA: 10 mg L^−1^; temperature: 15 °C; pH: 7.2).

HA degradation by different Ti–C dosages is shown in Fig. 6. As illustrated in Fig. 6, the HA removal rate increased with the increment in Ti–C amount added. The removal rate increased *ca.* 24.1% when the Ti–C amount increased from 0.5 g L^−1^ to 1.5 g L^−1^, and the removal rate further slightly increased *ca.* 3.5% when the Ti–C dosage changed from 1.5 g L^−1^ to 2.0 g L^−1^. The increase in the amount of Ti–C added into the solution would increase particle density in the illumination region, and produce more electron–hole pairs in the photocatalytic system, resulting in an increase in the number of active sites and more active substances, thereby improving the photocatalytic efficiency. However, at a higher Ti–C concentration, excessive catalyst particles would cause a shielding effect on lighting and hinder the light utilization on photocatalysts. Furthermore, aggregates would be formed easily by a large number of Ti–C particles, which was unfavorable to HA adsorption and light utilization.^20^

### 3.3 Characterization

The surface morphology and elemental composition of biochar and Ti–C were observed and determined using a scanning electron microscope (SEM) and an energy-dispersive spectrometer (EDS), respectively.

**Fig. 7:**
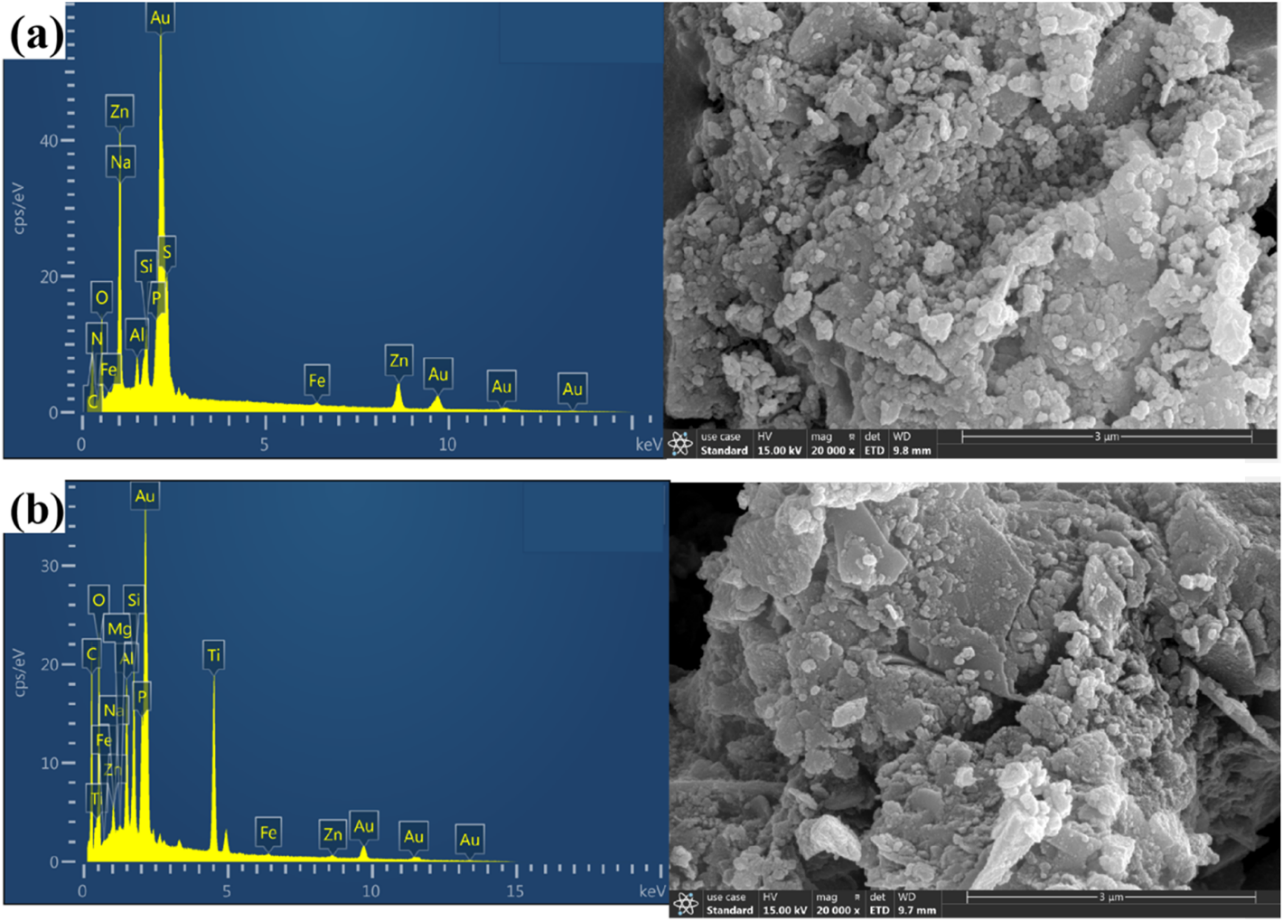
SEM micrographs and EDS analysis of biochar (a) and Ti–C (b).

As observed from Fig. 7, abundant numbers of crevices, pores and cavities were present on the surface of biochar. An irregularity with much sags and crests was displayed on Ti–C due to the formation of titanium dioxide crystalline on the carbon surface.^33–35^ There were certain cracks and micropores in the layer. The cracks were caused by shrinkage of the catalyst during drying and calcination, while micropores were formed by gas expansion and wall breaking during calcination. Such a structure enlarged Ti–C's specific surface area, and was conducive to HA adsorption on the product. Additionally, the effective area of receiving shining light on the composite was increased to produce more active free radicals and improved the catalytic activity of Ti–C. EDS analysis (Table 2) showed that the content of carbon increased 26.6% on the surface of TiO_2_ after modification, which indicated effective C-doping of the composite.

**Table 2:** EDS analysis of biochar, TiO_2_ and Ti–C

Element	Weight percentage (%)		
	Biochar	Titanium dioxide	Ti–C
C	22.85	3.76	29.94
N	2.15	0	0
O	14.57	35.63	25.57
Na	2.53	0	0.41
Al	2.11	0	5.30
Si	3.70	0	5.56
P	4.17	0	4.60
S	13.47	0	0
Fe	1.27	0	0.60
Zn	33.14	0	2.05
Ti	0	60.61	25.79

**Fig. 8:**
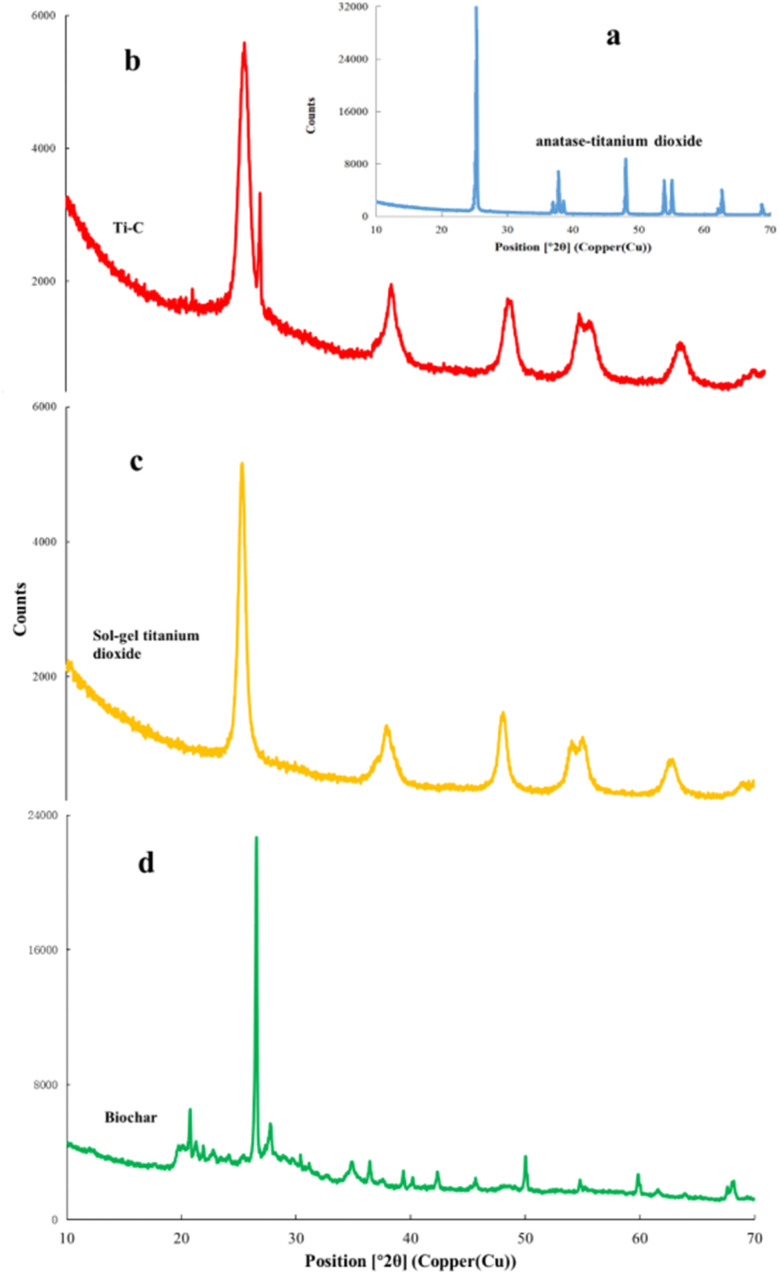
X-ray diffraction analysis of commercial anatase-TiO_2_ (a), Ti–C (b), synthesized TiO_2_ (c) and biochar (d).

X-ray diffraction (XRD) analysis was used for the determination of crystal structure of the material. XRD patterns of biochar, Ti–C and TiO_2_ are shown in Fig. 8. As shown in Fig. 8a, the characteristic peaks at 25.3, 37.9, 48.1, 54.0, 55.1, 62.7, and 68.8 were ascribed to the (101), (004), (200), (105), (211), (204), and (116) anatase crystal planes of a typical TiO_2_ (ref. 19) (JCPDS card #21-1272), respectively, which were all presented in our TiO_2_ synthesized by the sol–gel method (Fig. 8c). Moreover, in Fig. 8d, the characteristic peaks at 20.9, 26.6, 36.6, 39.5, 45.8, 50.2, and 60.0° were ascribed to the (100), (011), (110), (102), (021), (112), and (211) crystal planes of SiO_2_ (JCPDS card #79-1906) respectively on biochar.^25^Fig. 8b shows that the composed Ti–C prepared by the sol–gel method had obvious mentioned characteristic peaks of both SiO_2_ and TiO_2_, indicating a typical anatase structure. In addition, the Ti–C's characteristic peaks of SiO_2_ were weaker than those of biochar due to the formation of a titanium dioxide film on the surface of the product. XRD analysis indicated the successful synthesis of the Ti–C composite of TiO_2_ and biochar.

### 3.4 Photocatalytic mechanism

**Fig. 9:**
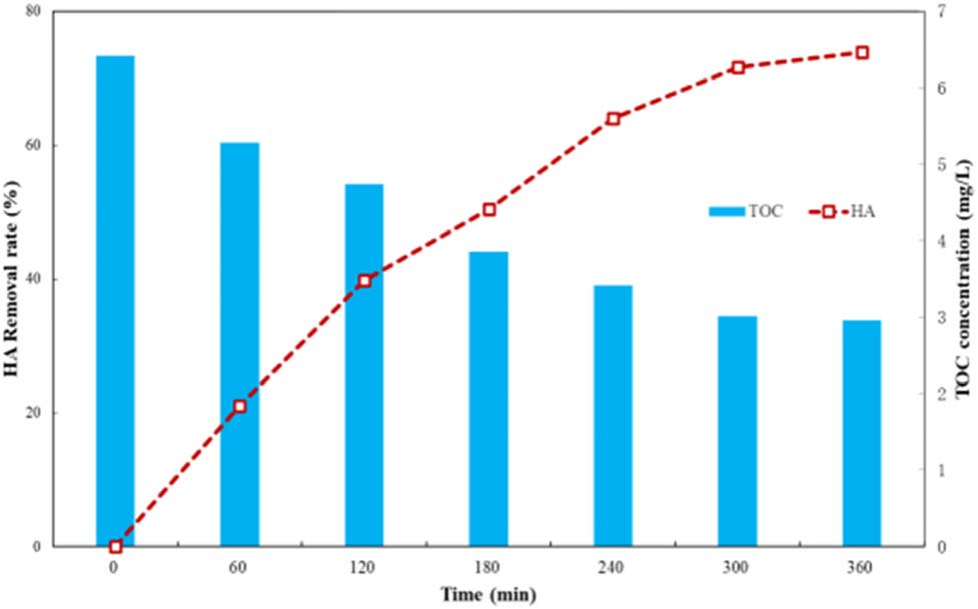
TOC change during HA degradation by Ti–C photocatalysis (HA: 10 mg L^−1^; TiO_2_: 1 g L^−1^; temperature: 15 °C; pH: 7.2).

**Fig. 10:**
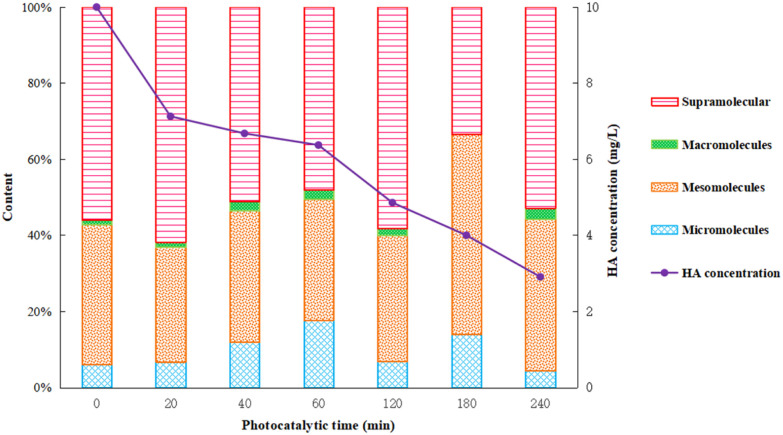
GC-MS analysis of HA degradation by Ti–C photocatalysis (HA: 10 mg L^−1^; TiO_2_: 1 g L^−1^; temperature: 15 °C; pH: 7.2).

After 40 minutes dark adsorption to reach equilibrium, the HA solution was filtered and sampled during the following 6 h photocatalysis process. The total organic carbon (TOC) content of the supernatant was determined using a TOC analyzer and the relative content of organic constituents by GC-MS. The results are shown in Fig. 9 and 10.

As shown in Fig. 9, changing the TOC concentration was consistent with the trend of HA degradation, which indicated that humic acid was finally mineralized into carbon dioxide and water by degradation.^19^ In the initial stage of the reaction, the TOC concentration in solutions was high, which indicated that degradation of humic acid by Ti–C generated a variety of intermediate products, and the overall degradation of the organic matters in water was slow and insignificant. The TOC concentration significantly reduced from macromolecular humic organic matters to small molecule intermediates under Ti–C photocatalytic effect, but not thoroughly converted into inorganic substances due to incomplete oxidization during this period. Thus, intermediate products played an important role in HA degradation, but little contribution on TOC removal. Those intermediates were hard to degrade within the limited reaction time, which hindered the contact between Ti–C and HA, and slowed down the photocatalytic degradation.^14^ The decomposition of organic matter was more complete with the proceeding of photocatalysis, thus the TOC concentration was low and HA removal was further increased and tended to be accomplished and stable after 5 h reaction.

Humic acid has a complex structure with a variety of active functional groups, such as high molecular weight part dominated by fat chain, and low molecule weight part including aromatic and polysaccharide.^14^ In this work, the specific components of humic acid were distinguished by molecular size, representing different types of organic compounds and the complexity of degradation. Information of the possible organic components in the solutions is given in Fig. 10. The smaller the molecule, the earlier the separation and appearance of peak time. The materials within the peak time of 0–5 min, 15–20 min, 21–29 min, and 30–32 min were defined as micro-molecules, meso-molecules, macro-molecules and supra-molecular parts, respectively, as presented in Fig. 10.

As shown in Fig. 10, the content of micro-molecules gradually increased with the reaction progress in the beginning 1 hour, and then had a downward trend after 1 hour. The content of meso-molecules remained constant in the first hour, and then increased gradually. The content of macro-molecules had been gradually accumulating during the whole photocatalysis. The content of supra-molecules decreased by degrees in the initial 1 hour and gradually increased after 1 hour. The results indicated that humic acid decomposed into complicated intermediate products at first and eventually converted into carbon dioxide and water under strong photocatalytic conditions.^36^ In the first hour, humic acid was quickly decomposed into various intermediates and then further degraded into final products, and thus, there were an increment in micro-molecules/meso-molecules and reduction in supra-molecules parts. As the Ti–C photocatalytic reaction proceeds, the intermediate products continuously accumulated, which led to a relatively slow degradation rate. Consequently, there was a decrement in the photocatalytic reaction rate and reduction of micro-molecules parts, but increasing content of meso-molecules and supra-molecules. Those parts were hard to be removed by synergic adsorptive or photocatalytic effect of Ti–C, and then turned to residuals in the solution after 4 h reaction.

**Fig. 11:**
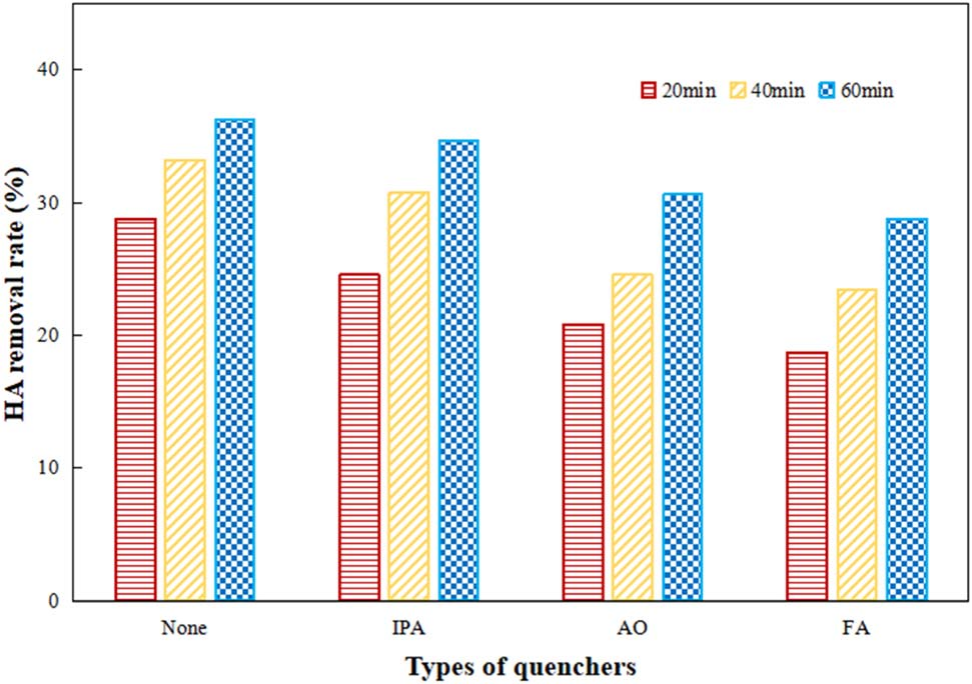
Quenching experiments of different scavengers on HA degradation (HA: 10 mg L^−1^; TiO_2_: 1 g L^−1^; temperature: 15 °C; pH: 7.2).

IPA, AO and FA were added as trappers for hydroxyl radicals, electron–holes and superoxide radicals to explore the effect of various free radicals during the photocatalytic process by Ti–C. IPA, AO and FA were added respectively for 60 min photocatalytic reaction, and the results are shown in Fig. 11.

As presented in Fig. 11, the photocatalytic activity was inhibited to different degrees after the addition of radical scavengers. The removal rates of HA with IPA, AO and FA were gradually decreased as the lighting time increased, which showed the obvious inhibitory effects of various active radicals. The inhibitory action of AO and FA was much stronger than that of IPA. After one hour reaction, HA degradation significantly decreased 20.7% by the addition of FA as compared to the initial one, while HA degradation was cut down 15.7% and 4.4% after the addition of AO and IPA respectively in comparison to the initial system. This indicated that superoxide radicals were the main active radicals and played an important role in efficient HA degradation; electron–holes and hydroxyl radicals also made certain contributions to HA decomposition during photocatalysis.^37^

## 4 Conclusions

Biochar-doped TiO_2_ (Ti–C) was proved to be effective towards the adsorptive and photocatalytic removal of humic acid from aqueous solutions simultaneously. HA photocatalytic removal by Ti–C was *ca.* 5% higher than simple TiO_2_ photocatalysis and *ca.* 20% higher than Ti–C adsorption. HA removal by Ti–C gradually increased and became stable around 72–74% (HA = 10 mg L^−1^, Ti–C = 1.0 mg L^−1^) after 6 h irradiation. Under an acidic condition (pH = 3.0), it was much favorable for humic acid photocatalytic degradation by Ti–C with *ca.* 50% removal efficiency, which was 27.8% higher than that at initial pH (7.2). A higher reaction temperature was conducive to HA photocatalytic degradation. The HA removal rate increased *ca.* 24.1% with the increase in added Ti–C amounts from 0.5 g L^−1^ to 1.5 g L^−1^, indicating that HA removal increased with the increment in Ti–C dosage.

Characterization analysis showed that Ti–C had a better adsorption capacity and more available area of irradiation, which was conducive to produce more active free radicals. TOC and GC-MS analysis verified that HA in water were degraded to carbon dioxide during persistent photocatalysis. Superoxide radicals were the main active radicals in efficient HA degradation in the system, while hydroxyl radicals and electron–holes also made certain contributions to HA decomposition. The composed biochar-modified titanium dioxide (Ti–C) showed great potential in HA removal from aqueous solutions in consideration of its practicability and economy efficiency.

## Data availability

The datasets used and/or analysed during the current study are available from the corresponding author on reasonable request.

## Author contributions

All authors contributed to the study conception and design. Guoqiao Wang participated in the preparation of materials, the conduct of experiments, the collection and analysis of data, and the writing of the manuscript. Jiawei Wang participated in the preparation of materials and the conduct of experiments. Xin Guo contributed to the process and the operation of the experiment and the writing of the manuscript. Yao Chen participated in the analysis of data, the process and the operation of the experiment, and contributed to the perfection of the manuscript.

## Conflicts of interest

All authors declare that they have no competing interests.

## Supplementary Material
